# Acceptability and Feasibility of Peer-to-Peer Text Messaging Among Adolescents to Increase Clinic Visits and Sexually Transmitted Infection Testing: Interrupted Times-Series Analysis

**DOI:** 10.2196/32416

**Published:** 2022-06-09

**Authors:** Marguerita Lightfoot, Joi Jackson-Morgan, Lance Pollack, Ayanna Bennett

**Affiliations:** 1 Division of Prevention Science Department of Medicine University of California San Francisco San Francisco, CA United States; 2 3rd Street Youth Center and Clinic San Francisco, CA United States

**Keywords:** HIV prevention, STI prevention, adolescents, youth, text messaging, SMS, peer-to-peer intervention, HIV, STI, HIV testing

## Abstract

**Background:**

Adolescents are disproportionately affected by sexually transmitted infections (STIs), including HIV. Many youths with asymptomatic STI or related symptoms do not seek treatment and may not be screened if accessing the health care system for other reasons.

**Objective:**

We examined intervention completion and changes in the number of new patients, the number of STI or HIV tests, and the sexual risk profile of patients over time to determine the feasibility and acceptability of a peer-driven text messaging strategy to connect youth to STI and HIV services.

**Methods:**

The intervention enlisted consecutive patients at an adolescent medicine clinic to send a text message to 5 peers they believed were sexually active and lived in the clinic’s service area. The intervention was evaluated using an interrupted time-series design in which baseline clinic service levels were documented during a 35-week lead-in period, followed by a 20-week intervention implementation period, and a 16-week period of continued clinic observation. Clinic and patient data were obtained through chart abstraction from intake forms that occurred during the entire study period. Analyses conducted in 2015 used a generalized linear mixed model.

**Results:**

Of the 153 patients approached to participate, 100 agreed to send SMS text messages. Most (n=55, 55%) reported no concerns with sending the text message. No adverse events or negative outcomes were reported. Adolescent STI testing, positive test results, and reported risk behavior increased post intervention, although this was not statistically significant, likely because of the small sample size.

**Conclusions:**

Given low youth uptake of health care services, and STI/HIV screening, in particular, new strategies are needed to address access barriers. Common approaches for reaching youth are resource-intensive and often miss those not connected to school or community programs. The peer-based text messaging strategy showed promise for both increasing the number of youths accessing health services and finding youths engaging in sexual risk behaviors and most in need of sexual health screening and services.

## Introduction

Adolescents are disproportionately affected by sexually transmitted infections (STIs). The World Health Organization estimates that 50% of the 30 million HIV infections worldwide occurred in youths between the ages of 15 and 24 years [1]. In the United States, almost one-quarter of new HIV infections are among youth and only 22% of sexually active youth have tested for HIV [2]. Furthermore, approximately half of all cases of STIs occur in people between the ages of 15 and 24 years [3]. However, many youths have asymptomatic STIs or do not seek services when they do have symptoms [4-6]. Youths who do access the health care system may not be screened for HIV [4,7]. Thus, despite the need for STI and HIV services, adolescents are among the most medically underserved.

Adolescents face barriers to accessing sexual health services, including stigma, low knowledge about STIs and available services, cost, clinic wait times, scheduling conflicts, and embarrassment [8-11]. Further, adolescents are least likely to have access to health care in the United States [12-14]. Hence, there has been a call for innovative solutions to increase adolescent STI/HIV screening [15]. In overcoming barriers to STI/HIV testing and access to health care, a peer-driven model may be particularly effective. Peers have the unique advantage of speaking from their own experiences; they also have credibility in delivering messages, as well as linguistic and cultural familiarity [16] and access to those in their social and sexual networks who are disconnected from the health care system. Peer education and outreach strategies have been successful in increasing the use of health resources in adolescents [17]. Further, SMS text messaging has proven successful in engaging STI/HIV screening among youth [18-20]. A recent systematic review found that text message reminders were most successful in increasing rates of HIV testing compared to other strategies [21]. We designed a peer-based approach that used text messaging to increase STI/HIV screening among adolescents. We report the feasibility and acceptability of this strategy.

## Methods

### Study Design

We used an interrupted time-series design to examine the impact of the text message intervention, deployed in an adolescent medicine clinic serving youth aged 12 to 24 years. Baseline service levels were documented during a 35-week lead-in period (weeks 1-35). This was followed by a 20-week period when patients were asked to send text messages to 5 friends who they believed were sexually active and lived in the clinic’s service area (weeks 36-55). Following the intervention was a 16-week period of continued clinic observation (weeks 56-72). Chart abstraction from intake forms continued during the entire study (weeks 1-72).

### Ethics Approval

The study was approved by the University of California, San Francisco, Institutional Review Board (approval #12-08516).

### Data Collection

Our target was 100 participants to ascertain feasibility and acceptability. Of the 153 patients approached by research staff, 28 (18%) refused, 25 (16%) did not have a mobile phone with them, and 100 (65%) provided informed consent and sent text messages. Participants were told that the goal of the text messages was to encourage their friends to visit the clinic and receive STI screening. Participants were provided a text messaging guide that reviewed considerations for developing messages (eg, let your friend know you care about them). Participants developed their own message. Subsequent to sending the messages, participants were provided US $10 for completing a short posttexting interview. The text message and responses from friends while the participant was still in the clinic were recorded by research staff. Staff recontacted participants 1 week after their clinic visit to ascertain issues with friends following the intervention (n=93).

### Data Analysis

Analyses compared the 35-week “pretexting” data to the 36-week postintervention period. Additional comparisons accounted for seasonal fluctuations in service levels by comparison to the same dates in the preceding year. We examined changes in the number of new patients seen (including patients not seen for at least 12 months), the number of STI tests conducted and positive results, the number of HIV tests conducted and positive results, and the number of at-risk patients seen at the clinic. Patients were characterized as at risk if they reported multiple partners, infrequent condom use, or had an STI at intake. Counts for each outcome were aggregated into average monthly totals, and analyses were performed using a generalized linear mixed model. Outcomes were modeled as overdispersed Poisson distribution variables. For clarity, only the mean of the counts during each time period was provided.

## Results

### Peer Texting

All participants sent the required 5 messages (see Textbox 1 for examples of messages sent and received), and 18% (n=18) of participants sent more than 5 messages. In the brief posttexting interview, most patients (n=55, 55%) reported no concerns with sending the text message, 18% (n=18) expressed worry about their friends’ response, and 27% (n=27) expressed concern that their friend would not heed their advice (eg, their friend would not call the clinic or would not listen). In the 1-week postmessaging interview, no youth reported adverse events or negative outcomes from sending the message.

**Examples of text messages sent and received by participants.**

**Example messages sent by participants**

- “Hey girl this _____________ wen u cum bck frm Texas here at the clinic on [redacted] u shuld stp bi they hav gud classes n program n ull fel lyk home”
- “Hey. So I’m at the clinic and I’m gonna get tested. You should come get checked too. I’ll come with you if you want. It’s over on [redacted] next to the new WingStop.”
- “Hey I’m at the [redacted] it’s a good idea to get checked out”
- “I just got checked out at the [redacted] and they were really nice they offer free testing and helped me a lot, you should come down too.”

**Example messages received from peers**

- “I just got check, that shit ain’t nothing to be played with”
- “Lmfao. Nice. Okay well when I get home”
- “Fa sho. What time it ends? I get out @ 4”
- “Bitch what you telling me for? I go to my dr. every 3 months for the free and I damn sho aint got HIV”

### Clinic Attendance and STI/HIV Testing

A total of 430 new patients were seen at the clinic during the 72 weeks of data collection, of whom 338 (79%) were female. New patients were 61% (n=262) African American and 17% (n=73) Hispanic, had a mean age of 19.6 (SD 2.6, range 13-25) years, and primarily identified as heterosexual (n=375, 87%). Almost half of the patients reported they had no regular doctor (n=185, 43%) or health care (n=194, 45%).

In a brief posttexting interview with the 100 participants, most patients (n=55, 55%) reported no concerns with sending the text message, 18% (n=18) expressed worry about their friends’ response, and 27% (n=27) expressed a concern that their friend would not heed their advice. In a follow-up interview 1 week after messaging, no youth reported adverse events or negative outcomes from sending the message.

We examined changes in the number of new patients seen, the number of STI tests conducted and positive results, the number of HIV tests conducted and positive results, and the number of at-risk patients seen at the clinic. Patients were characterized as at risk if they reported multiple partners, infrequent condom use, or any STI infection. All clinic services outcomes increased during the posttexting period compared to the pretexting period (Table 1), including weekly STI tests (4.11 vs 5.18, *P*=.58), weekly number of youths testing positive for STI (0.60 vs 0.76, *P*=.17), weekly number of youths tested for HIV (0.91 vs 1.35, *P*=.48), and weekly number of patients reporting high-risk sexual behaviors (4.89 vs 5.29, *P*=.55). However, these findings did not achieve statistical significance. In order to account for possible seasonal and period effects, we also examined the means for a subset of the preintervention phase that matched the calendar months covered in the postintervention phase but occurred 1 year earlier. Findings were similar, suggesting that the variation in average patient counts between the pre- and postintervention periods was not due to seasonal variation.

**Table 1:** Average weekly patient count during the preintervention, intervention, and postintervention phases.

Variable	Weekly patient count^a^, mean (SD)			
	Preintervention (weeks 1-35)	Calendar-matched preintervention (weeks 6-23)	Intervention (weeks 36-55)	Postintervention (weeks 56-72)
Total	6.00 (3.25)	5.50 (2.83)	5.40 (2.11)	6.59 (2.79)
Tested for STI^b^	4.11 (2.86)	3.50 (2.50)	3.95 (2.16)	5.18 (2.13)
Positive for STI	0.60 (.081)	0.39 (0.61)	0.60 (0.88)	0.76 (0.90)
Tested for HIV	0.91 (1.72)	0.50 (0.71)	0.85 (0.88)	1.35 (1.69)
High sexual risk^c^	4.89 (2.80)	4.28 (2.32)	4.15 (1.95)	5.29 (2.34)

^a^Count of new patients and re-engaged patients (patients not seen for at least 12 months).

^b^STI: sexually transmitted infection.

^c^Self-reported as having ≥2 sex partners in the prior 3 months, or self-reported an STI diagnosis in the past year, or tested positive for gonorrhea or chlamydia at the clinic visit, or self-reported not always using a condom when having sexual intercourse.

## Discussion

### Key Findings

Given the low number of youths accessing health care services and STI/HIV screening, new strategies are needed to address the barriers that exist. We found that text messaging between peers is a feasible and acceptable strategy with potential for increasing STI/HIV testing. We did not provide standard messages; rather, we were interested in whether youth were able to develop their own persuasive messages. Most youths agreed to send a message and did not express concern or experience complaints from peers. One in 5 participants sent more than the required number of messages. The approach appears feasible and acceptable. The simplicity and low-resource requirements of this approach also make it more sustainable and significantly less costly than recent efforts to build smartphone apps, social media campaigns, or other technology-based interventions to increase testing [18].

We were unable to examine if messages were read or to directly ask recipients about their reactions. Given that over 97% of text messages are opened and 90% are read within 3 minutes of their delivery [22], we did record the immediate response from message recipients. There were few negative text responses, and no negative consequences or adverse events reported by participants during the follow-up interviews. This suggests that the intervention may also be acceptable to the recipients of the message.

### Limitations

This study was a proof of concept, and, thus, has limitations in interpretation. The study had a small sample size and was conducted at a single adolescent medical clinic. There may have been selection bias where youths who were more comfortable discussing sexual health with their friends may be overrepresented. However, it is likely that a formal implementation of this intervention would also be on an opt-in basis, strengthening the external validity of our pilot.

### Conclusions

While this pilot study was not powered to detect significant differences over time, we did find encouraging increases in the number of weekly STI tests conducted, the number of youths testing positive monthly for STIs, the number of youths tested monthly for HIV, and the number of patients reporting sexual risk behaviors. Thus, text messaging may have increased the number of youths accessing services and STI/HIV screening and reached those engaging in risky sexual behavior who are most in need of screening. This is particularly noteworthy given the clinic primarily serves a low-income population of Black and Hispanic adolescents, a population disproportionately impacted by STI and HIV [23].

## Abbreviations

STI: sexually transmitted infection
